# Early use of corticosteroids in non-critical patients with COVID-19 pneumonia (PREDCOVID): a structured summary of a study protocol for a randomised controlled trial

**DOI:** 10.1186/s13063-021-05046-6

**Published:** 2021-01-26

**Authors:** Mauricio Salinas, Paulette Andino, Leonor Palma, Javiera Valencia, Elizabeth Figueroa, Jhonatan Ortega

**Affiliations:** 1 Facultad de Medicina, Universidad de Chile, Santiago, Chile; 2 Instituto Nacional del Tórax, Santiago, Chile; 3 Hospital Santiago Oriente, Santiago, Chile; 4 Universidad de Los Andes Facultad de Medicina, Santiago, Chile

**Keywords:** COVID-19, Randomised controlled trial, protocol, pragmatic clinical trial, prednisone

## Abstract

**Objectives:**

To evaluate the efficacy of early treatment with prednisone to decrease the progression of COVID-19 pneumonia.

**Trial design:**

This is a pragmatic, non-blinded, randomized, two arms, parallel trial.

**Participants:**

Patients between 18 and 90 years, with COVID-19 pneumonia, confirmed by RT PCR. The setting for the trial is the Hospital Santiago Oriente which is a secondary level hospital with an emergency room, intensive care, and all basic specialties of medicine.

Inclusion Criteria:

- 18 years or more
- COVID-19 confirmed by RT-PCR
- Oxygen requirements up to 35% by venturi mask or 5 liters per minute by nasal cannula (approximately FiO2 40%)
- Consent form signed

Exclusion Criteria:

- Previous steroid use for more than 48 hours.
- Pregnancy
- Chronic respiratory failure
- Requirements of mechanical ventilation (invasive or no invasive)
- Chronic liver damage Child Pugh B or C
- Chronic kidney disease stage IV or V.
- Immunosuppressed
- Participation in another trial.

**Intervention and comparator:**

Experimental arm

Prednisone 40 mg days 1 to 4. Then Prednisone 20 mg days 5 to 8. Usual care defined by the attending physician.

Control arm

No intervention. Usual care defined by the attending physician.

**Main outcomes:**

Primary outcome

Composite Primary End-point: Admission to ICU, Need for Invasive Mechanical Ventilation or All-cause Death by Day 28

Secondary outcomes (followed until day 28).

- Time to respiratory deterioration
- Incidence of patients requiring mechanical ventilation:
- Number of days on mechanical ventilation

Special emphasis will be placed on observing the following serious adverse events

- Deterioration of the glycemic profile that requires the use of insulin
- Delirium
- Incidence of hospital infections (pneumonia, urinary tract infection, device associated infections)
- Cumulative incidence of grade 3 and 4 adverse events (AE).
- Interruption or temporary suspension of treatment for any reason

**Randomisation:**

Randomisation in permuted block. Computer generated random numbers in an allocation rate of 1:1. Stata 14.0 was used.

Allocated by the principal investigator (direct communication).

**Blinding (masking):**

Patients not blinded.

Caregivers not blinded.

Participants not blinded.

Statistician will not know the allocation.

**Numbers to be randomised (sample size):**

92 patients in each arm.

184 total number of patients.

**Trial Status:**

Protocol version 2.0., approved October 2, 2020.

Trial ongoing.

Recruitment start: June 23, 2020.

Anticipate finish recruiting: November 30, 2020.

The protocol has been submitted before the last patient and last visit. The delay in sending to publication is responsibility of the authors.

**Trial registration:**

Early Use of Corticosteroids in Non-critical Patients With COVID-19 Pneumonia (PREDCOVID). Registration number NCT04451174. Date of trial registration: June 26, 2020.

**Full protocol:**

The full protocol is attached as an additional file, accessible from the Trials website (Additional file 1). In the interest in expediting dissemination of this material, the familiar formatting has been eliminated; this Letter serves as a summary of the key elements of the full protocol.

**Supplementary Information:**

The online version contains supplementary material available at 10.1186/s13063-021-05046-6.

## Supplementary Information

**Additional file 1.** Full study protocal.

## Data Availability

Data will be available from the author on reasonable request. Please contact mrsalinas@uchile.cl

