# Supplementary material for: Early use of corticosteroids in non-critical patients with COVID-19 pneumonia (PREDCOVID): a structured summary of a study protocol for a randomised controlled trial
Source: Trials. 2021 Jan 26;22:92. doi: 10.1186/s13063-021-05046-6 (PMC7835442; doi:10.1186/s13063-021-05046-6)
Supplement: Supplementary file 1 — Additional file 1. Full study protocal. [file 13063_2021_5046_MOESM1_ESM.docx]

**Title**

Early Use of Corticosteroids in Non-critical Patients With COVID-19 Pneumonia (PREDCOVID)

**Background:**

We are in the middle of the coronavirus pandemic, facing in Chile, an increasing number of cases and deaths. As of June 11, 2020 there are 154092 cases confirmed by COVID – 19 in our country and 2648 deaths ^1.^.

Most patients have mild illness, but older people and those with comorities can develop severe illness that requires hospitalization, some form of ventilatory support, and need to enter an intensive care unit. Pathopathology occurs in two distinct overlapping phases, the initial pathogenic with viral replication, followed by the inflammatory response of the host with varying degrees of severity associated with different clinical characteristics. Pathological progression in some cases of severe COVID-19 would be explained by excess pro-inflammatory cytokines which leads to diffuse alveolar damage, with the development of acute respiratory distress syndrome (DRA) and inflammatory commitment of multiple systems up to death ^2.3.^.

In the absence of any proven antiviral therapy, current clinical treatment is mainly supportive care, supplemental oxygen and mechanical ventilatory support.

Clinical trials have been published and convened to demonstrate the usefulness of therapies in the context of this pandemic and to favor this, over the use of non-protocolized therapies without evidence^4.^.

The role of corticosteroids is not fully defined. Observational studies report better results in decreased disease progression in those COVID-19 patients who received corticosteroids early  ^5.6.^.

We postulate in this study that treatment in moderate disease (defined as one that requires supplemental oxygen, but without the need for ventilatory support) can attenuate the host's excessive respiratory and systemic inflammatory responses.

The goal of this study is to evaluate the effectiveness of early corticosteroid treatment to decrease the progression of moderate to severe disease.

**Design:**

This study is a controlled, randomized, non-blind clinical trial to evaluate the efficacy and efficacy of corticosteroid use in hospitalized adult patients diagnosed with COVID-19 with moderate disease. It is a pragmatic trial

**Site**

The study will be carried out at the Hospital Santiago Oriente. It is a medium complexity hospital. The hospital has basic medical specialties (pediatrics, obstetric gynecology, internal medicine and surgery), an emergency unit, operating room and critical care unit.

**Inclusion criteria:**

Inpatients eligible for inclusion:

1. 18 years of age or older,
2. Confirmed case of COVID-19 is defined as a patient with respiratory symptoms of COVID19 plus positive PCR in nasopharyngeal sample or CT scan with characteristic opacity. Cases with characteristic CT will be confirmed later with serology or PCR.
3. Oxygen requirements of up to 5 lt per narcotic or 35% per Venturi mask that is not for pre-existing disease.
4. Signed informed consent.

**Exclusion criteria:**

1. Patient in treatment with corticosteroids for 30 days or more.
2. Pregnant woman
3. Patients in Invasive or non-invasive mechanical ventilation.
4. Chronic kidney disease stage IV or higher.
5. Chronic liver damage Child Pugh B or C.
6. Immunosuppressed.
7. Chronic respiratory failure-
8. Have previously received corticosteroids for 48 hours or more
9. Participation in another protocol.

**Primary outcome:**

Admission to critical patient unit, need for mechanical ventilation or death at 28 days.

Several secondary outcomes, until 28 days, with exploratory intent will also be evaluated.

- Time for respiratory deterioration
- Incidence of patients requiring Mechanical Ventilation:
- Number of days in mechanical ventilation
- Number of days

Emphasis on observing the following serious adverse events

- Deterioration of the glycemic profile requiring insulin use
- Delirium
- Incidence of intrahospital infections (pneumonia, urinary tract infection, device-associated infections)
- Cumulative incidence of grade 3 and 4 adverse events (EA).
- Interruption or temporary discontinuation of treatment for any reason

The data will be obtained in a standardized case report form including demographic data, information on clinical symptoms or signs in the presentation, laboratory, and radiological results during admission.

All laboratory tests and radiological evaluations, including simple chest x-ray and chest CT, will be performed at the discretion of the treating physician.

**Intervention:**

- Intervention group

Patients will receive a course of corticosteroids with prednisone 40 mg oral per day for 4 days and then 20 mg daily for 4 days.

Treatment will be initiated before the 7th day of hospitalization.

- Control group

They will not receive corticosteroids.

They will receive standard care according to national and local recommendations.

All should receive thromboprophylaxis with low molecular weight heparins according to international recommendations.

All patients will receive omeprazole in doses of 20 mg oral or famotidine 40 mg oral.

The use of antibiotics is at the discretion of the treating physician.

The co-interventions are not regulated by the protocol, but a record will be kept of them for later analysis.

**Sample size estimates**

Based on a previous publication study that showed a decrease in the frequency of the same primary outcome in this study from 44% to 23%, with a power of 80% and a significance level of 5% to two tails, an n of 88 patients per branch is estimated necessary, to which we will add 5% for possible losses , giving a total of 92 patients per branch.

The sample will be random in permuted blocks

**Assignment of the intervention.**

The random blocks will be known only to the principal investigator (MS) who will oversee informing directly (telephone) to the field investigators, which treatment corresponds.

**Statistical analysis proposed.**

A description of means and standard deviation will be made for quantitative variables and absolute and relative frequencies for qualitative variables.

Fisher's exact or square chi test will be tested for the primary outcome analysis.

No imputation of lost data will be made.

Exploratory analyses will also be performed for secondary outcomes with cox proportional hazard model, Kaplan meier curves, log rank testing, and multivariate models based on the results found.

All the analyses will be performed with principle of intention to treatment.

Statistical significance tests will be performed with an alpha of 0.05, two tiles.

The analysis will be carried out by a statistician who does not participate in the study and does not know the treatment received by each patient.

**Follow up**

| **Exam** | **Day 1** | **Day 3** | **Day 5** | **Day 7** |
| --- | --- | --- | --- | --- |
| Blood count. | X | X | X | X |
| PCR. | X | X | X | X |
| Ferritin. | X |  |  |  |
| Pulse oximetry. | X | X | X | X |
| Respiratory rate. | X | X | X | X |
| Blood pressure. | X | X | X | X |
| Blood glucose. | X | X | X | X |
| Hepatic P. | X | X | X | X |
| Creatinine / GOOD. | X | X | X | X |
| Delirium surveillance*. | X | X | X | X |

* Rating according to the following guideline: comma, confusing, normal, agitated.

The need for image control (chest x-ray or chest CT) is at the discretion of the treating physician).

At day 28 will be assessed:

- Vital status

- Mechanical ventilation status

- Days hospitalizacion

- Other infections (pneumonia, urinary tract infections, device infection)

- Mental status

- Other complications reported.

**Ethical aspects.**

For the inclusion of patients, written informed consent will be requested.

In the actual epidemiology context, there is restrictions on family visits and risk of COVID-19 spread. So, if the patient cannot sing the consent, a request will not be made to family members.

The study will be carried out according to the principles of the Declaration of Helsinki and was approved by the local ethics committee.

**References**

1. Confirmed cases in Chile COVID-19 - Ministry of Health - Government of Chile [Internet]. [cited 2020 Jun 13]. Available from: https://www.minsal.cl/nuevo-coronavirus-2019-ncov/casos-confirmados-en-chile-covid-19/

2. Berlin DA, Gulick RM, Martinez FJ. Severe Covid-19. N Engl J Med. 2020 May 15;

3. Mehta P, McAuley DF, Brown M, Sanchez E, Tattersall RS, Manson JJ, et al. COVID-19: consider cytokine storm syndromes and immunosuppression. Lancet. 2020;395:1033–4.

4. Rome BN, Avorn J. Drug Evaluation during the Covid-19 Pandemic. N Engl J Med. 2020 Apr 14;

5. Early Short Course Corticosteroids in Hospitalized Patients with COVID-19 Clinical Infectious Diseases Oxford Academic [Internet]. [cited 2020 Jun 13]. Available from: https://academic.oup.com/cid/advance-article/doi/10.1093/cid/ciaa601/5840526

6. Methylprednisolone for Patients With COVID-19 Severe Acute Respiratory Syndrome - Study Results - ClinicalTrials.gov [Internet]. [cited 2020 Jun 13]. Available from: https://clinicaltrials.gov/ct2/show/results/NCT04323592?view=results

Letter to the editor: COVID-19 study protocol structured summary template

**Title**

Early Use of Corticosteroids in Non-critical Patients With COVID-19 Pneumonia (PREDCOVID): A structured summary of a study protocol for a randomised controlled trial

**Authors**

Mauricio Salinas, MD. Facultad de Medicina, Universidad de Chile. Mrsalinas@uchile.cl

Paulette Andino, MD. Instituto Nacional del Tórax. Chile.

paulette.a.sarmiento@gmail.com

Leonor Palma RN. Hospital Santiago Oriente, Chile.

[leo.palma@profesor.duoc.cl](mailto:leo.palma@profesor.duoc.cl)

Javiera Valencia MD. Universidad de Los Andes Facultad de Medicina, Chile.

[jfvalencia@miuandes.cl](mailto:jfvalencia@miuandes.cl)

Elizabeth Figueroa MD. Universidad de Los Andes Facultad de Medicina, Chile.

[Eli.figueroa24@gmail.com](mailto:Eli.figueroa24@gmail.com)

Jhonatan Ortega MD. Hospital Santiago Oriente, Chile.

[jonatan11ortega@yahoo.it](mailto:jonatan11ortega@yahoo.it)

Abstract

**Objectives**

To evaluate the efficacy of early treatment with prednisone to decrease the progression of COVID-19 pneumonia.

**Trial design**

This is a pragmatic, non-blinded, randomized, two arms, parallel trial.

**Participants**

Patients between 18 and 90 years, with COVID-19 pneumonia, confirmed by RT PCR. The setting for the trial is the Hospital Santiago Oriente which is a secondary level hospital with an emergency room, intensive care, and all basic specialties of medicine.

Inclusion Criteria:

18 years or more

COVID-19 confirmed by RT-PCR

Oxygen requirements up to 35 % by venturi mask or 5 liters per minute by nasal cannula (approximately FiO2 40%)

Consent form signed

Exclusion Criteria:

Previous steroid use for more than 48 hours.

Pregnancy

Chronic respiratory failure

Requirements of mechanical ventilation (invasive or no invasive)

Chronic liver damage Child Pugh B or C

Chronic kidney disease stage IV or V.

Immunosuppressed

Participation i n another trial.

**Intervention and comparator**

Experimental arm

Prednisone 40 mg days 1 to 4. Then Prednisone 20 mg days 5 to 8. Usual care defined by the attending physician.

Control arm

No intervention. Usual care defined by the attending physician.

**Main outcomes**

Primary outcome

Composite Primary End-point: Admission to ICU, Need for Invasive Mechanical Ventilation or All-cause Death by Day 28

Secondary outcomes (followed until day 28).

• Time to respiratory deterioration

• Incidence of patients requiring mechanical ventilation:

• Number of days on mechanical ventilation

Special emphasis will be placed on observing the following serious adverse events

• Deterioration of the glycemic profile that requires the use of insulin

• Delirium

• Incidence of hospital infections (pneumonia, urinary tract infection, device associated infections)

• Cumulative incidence of grade 3 and 4 adverse events (AE).

• Interruption or temporary suspension of treatment for any reason

**Randomisation**

Randomisation in permuted block. Computer generated random numbers in an allocation rate of 1:1. Stata 14.0 was used.

Allocated by the principal investigator (direct communication).

**Blinding (masking)**

Patients not blinded.

Caregivers not blinded.

Participants not blinded.

Statistician will not know the allocation.

**Numbers to be randomised (sample size)**

92 patients in each arm.

184 total number of patients.

**Trial Status**

Protocol version 2.0., approved October 2, 2020.

Trial ongoing.

Recruitment start: June 23, 2020.

Anticipate finish recruiting: November 30, 2020.

The protocol has been submitted before the last patient and last visit. The delay in sending to publication is responsibility of the authors.

**Trial registration**

Early Use of Corticosteroids in Non-critical Patients With COVID-19 Pneumonia (PREDCOVID)

Registration number NCT04451174 (<https://clinicaltrials.gov/ct2/show/NCT04451174>)

Date of trial registration: June 26, 2020.

**Full protocol**

The full protocol is attached as an additional file, accessible from the Trials website (Additional file 1). In the interest in expediting dissemination of this material, the familiar formatting has been eliminated; this Letter serves as a summary of the key elements of the full protocol.

**Keywords**

COVID-19, Randomised controlled trial, protocol, pragmatic clinical trial, prednisone.

**Declarations**

**Ethics approval and consent to participate**

Approved by the Scientific Ethical Committee of the Servicio de Salud Metropolitano Oriente on June 16, 2020 under the title “Uso precoz de corticoides en pacientes

hospitalizados con enfermedad moderada por COVID -19 (PREDCOVID)”.

Statement

I certify that this trial has received ethical approval from the referred ethical committee.

Consent to participate is mandatory for all participants in this trial

**Consent for publication.** Not applicable.

**Availability of data and materials**

Data will be available from the author on reasonable request. Please contact [mrsalinas@uchile.cl](mailto:mrsalinas@uchile.cl)

**Competing interests**

The authors declare that they have no competing interest

**Funding**

The study is not funded.

**Authors' contributions**

MS. Conception. Design of work.

PA. Conception. Design of work. Organization and supervising work team

LP. Organization and supervising work team. Collect all patient’s information and check it.

JV. Obtain Consent. Follow up of patients. Collect patient information.

EF. Obtain Consent. Follow up of patients. Collect patient information.

JO. Obtain Consent. Follow up of patients. Collect patient information.

All of the authors have revised and approved the protocol.

**Acknowledgements**

Not applicable
